# Outbreaks of Pandemic (H1N1) 2009 and Seasonal Influenza A (H3N2) on Cruise Ship

**DOI:** 10.3201/eid1611.100477

**Published:** 2010-11

**Authors:** Kate A. Ward, Paul Armstrong, Jeremy M. McAnulty, Jenna M. Iwasenko, Dominic E. Dwyer

**Affiliations:** Author affiliations: New South Wales Health, Sydney, New South Wales, Australia (K.A. Ward, J.M. McAnulty);; Western Australian Department of Health, Perth, Western Australia, Australia (P. Armstrong);; South Eastern Area Laboratory Services, Sydney (J.M. Iwasenko);; Institute of Clinical Pathology and Medical Research, Sydney (D.E. Dwyer)

**Keywords:** Influenza A virus, subtype H1N1, pandemic, pandemic (H1N1) 2009, disease outbreaks, population health surveillance, influenza, cruise ship, viruses, research

## Abstract

To determine the extent and pattern of influenza transmission and effectiveness of containment measures, we investigated dual outbreaks of pandemic (H1N1) 2009 and influenza A (H3N2) that had occurred on a cruise ship in May 2009. Of 1,970 passengers and 734 crew members, 82 (3.0%) were infected with pandemic (H1N1) 2009 virus, 98 (3.6%) with influenza A (H3N2) virus, and 2 (0.1%) with both. Among 45 children who visited the ship’s childcare center, infection rate for pandemic (H1N1) 2009 was higher than that for influenza A (H3N2) viruses. Disembarked passengers reported a high level of compliance with isolation and quarantine recommendations. We found 4 subsequent cases epidemiologically linked to passengers but no evidence of sustained transmission to the community or passengers on the next cruise. Among this population of generally healthy passengers, children seemed more susceptible to pandemic (H1N1) 2009 than to influenza (H3N2) viruses. Intensive disease control measures successfully contained these outbreaks.

## Abstract 2

Although pandemic virus spread rapidly, especially among children, intensive control measures successfully contained these outbreaks.

During April 2009, pandemic (H1N1) 2009 (pandemic influenza) virus began to circulate worldwide. In Australia, public health efforts were initially focused on delaying the entry of the virus into the country. By May 24, 2009, a total of 14 cases had been identified nationally, 2 in New South Wales (NSW), and all were associated with international travel.

On May 24, the Australian Quarantine Inspection Service reported that 6 passengers of a cruise ship had respiratory symptoms, and a point-of-care test showed positive influenza A virus results for all. The ship had departed from Sydney on a 10-day cruise in the Pacific Ocean on May 16 (cruise A) and stopped at 2 islands, neither of which had reported circulation of pandemic influenza virus. None of the sick passengers had been in countries known to be affected by this influenza strain in the week before boarding. Thus, with no reason to suspect that the pandemic strain was circulating on board, passengers were allowed to disembark in Sydney on May 25.

On May 25, the 4 available respiratory samples taken from sick passengers were quickly couriered to the South Eastern Area Laboratory Service (the major public health viral laboratory serving eastern Sydney) for influenza virus nucleic acid testing (NAT) by real-time reverse transcription­–PCR (RT-PCR). Of these 4 samples, 2 were positive for pandemic influenza virus and 2 were positive for influenza A (H3N2) (seasonal influenza) virus.

In response, NSW Health requested that all passengers (1,963 from Australia and 7 from elsewhere) who were experiencing influenza-like illness (ILI) isolate themselves from healthy persons and that all asymptomatic passengers quarantine themselves for 7 days after disembarkation (or 7 days after onset of symptoms if they developed). This advice was communicated to passengers on the day of disembarkation through media alerts, the NSW Health website, and telephone information lines. Subsequently, passengers were contacted by telephone to ensure that they understood containment measures (how to prevent virus spread). Oseltamivir treatment (75 mg 2×/d for 5 days) was recommended for passengers or crew members with ILI (defined as >2 of the following: cough, fever, runny nose, or blocked nose) within 48 hours of onset and oseltamivir prophylaxis (75 mg 1×/d for 10 days) for those in close contact with patients with laboratory-confirmed cases.

On May 25, all crew members were assessed for illness. Symptomatic members were isolated on shore, and the rest were given oseltamivir prophylaxis and continued to serve on the ship’s next voyage (cruise B), which departed later the same day. Cruise B traveled along the northern coast of Australia for 7 days and made a short stop at Brisbane before returning to Sydney on June 1. To minimize the risk for infection, enhanced cleaning regimens were conducted before cruise B, and NSW Health sent a public health doctor on the cruise to conduct intense surveillance for symptomatic passengers and crew.

Outbreaks of influenza have previously been reported on cruise ships (*1*–*6*), but the circumstances and extent of transmission have not been well documented. The cocirculation of pandemic and seasonal influenza viruses on cruise ship A provided a unique opportunity to compare symptoms, severity, and attack rates of pandemic and seasonal strains. We describe our outbreak investigation, compare the epidemiology of the 2 influenza virus subtypes, and explore effectiveness of control measures.

## Methods

### Case Definition

We defined a confirmed influenza A case as illness in a cruise A passenger in whom influenza A virus was detected by NAT during the cruise or within 7 days after disembarkation (regardless of symptoms). A case of pandemic influenza was defined as illness in a person with positive RT-PCR results for that virus. Further subtyping was conducted for 44 of 100 patients with positive influenza A but negative pandemic influenza virus results by NAT; all had positive results for seasonal influenza virus. Consequently, we defined a case of seasonal influenza as illness in a person with positive influenza A virus results by NAT but negative pandemic influenza virus results and in whom influenza subtyping for seasonal influenza virus by RT-PCR either produced positive results or was not conducted. A primary case was defined as illness in the first person in a cabin to report ILI symptoms; a co-primary case, as illness in a person who reported symptom onset within 24 hours after a primary case; and a secondary case, as illness in a person whose symptoms developed >24 hours after symptom onset in the primary case-patient. Case-patients were considered infectious for 24 hours before and 7 days after symptom onset. For the childcare center investigation, children who remained asymptomatic throughout the cruise were considered susceptible to influenza infection at each childcare session attended. Children in whom ILI developed were considered susceptible before the infectious period began.

### Case Detection

We obtained a list of the names, sex, dates of birth, nationality, contact details, and cabin numbers of all passengers and crew members on cruise A. We reviewed the cruise ship’s medical records to find passengers who had sought treatment for ILI during cruises A and B. Isolated symptomatic passengers from cruise A were referred to nearby hospitals for testing. Quarantined asymptomatic passengers were asked to report if symptoms developed; if so, laboratory testing was conducted. Crew members and passengers on cruise B were asked to immediately report fever or respiratory symptoms to medical staff and were tested for influenza by at least 2 point-of-care tests taken >24 hours apart. In all 8 Australian states and territories, public health legislation requires diagnostic laboratories to report confirmed influenza cases to the jurisdictional health department (*7*). The names of influenza case-patients reported after completion of cruise A were checked against the ship’s manifest.

### Data Collection

Because the investigation was part of a public health control initiative, formal ethics committee review was not required. Experienced public health staff interviewed case-patients at the time of diagnosis and used a standardized questionnaire to determine symptoms, hospitalization status, and oseltamivir use. This information was entered into a statewide database. Passengers who shared a cabin with case-patients who had pandemic influenza were also interviewed about respiratory symptoms. Laboratory testing initially focused on identifying pandemic influenza cases by using the specific RT-PCR; samples determined negative for pandemic influenza virus by NAT were tested for influenza A (including seasonal influenza virus) several weeks after passengers had disembarked.

Approximately 6 weeks after disembarking, all 50 passengers who had had pandemic influenza were reinterviewed about the duration and severity of their illness. These passengers included 3 interstate residents who had been treated in NSW (and excluded 28 non–NSW case-patients as a convenience sample) and the 45 NSW case-patients who had seasonal influenza (excluding 55 non-NSW case-patients and 17 NSW case-patients for whom test results were not available at the time of interview). Ultimately, 62 cases of seasonal influenza were identified among NSW passengers; complete symptom data from 50 passengers who were interviewed at the time of diagnosis were recorded in the statewide database.

### Childcare Center Investigation

On-board childcare activities were provided in 3 daily sessions (9:00 am­­­–10:00 pm) in 3 areas of the ship for 3 age groups: 3–6, 7–12, and >13 years of age. Because the pandemic outbreak appeared to begin in and primarily affect children 3–6 years of age, the epidemiologic investigation focused on this group. Most childcare activities for this group took place in 1 room. We examined childcare attendance records for this group and, ≈6 weeks after disembarkation, interviewed the parents of all children in this group about symptoms, vaccination history, and composition of the traveling group. All specimens collected from childcare attendees were tested for pandemic and seasonal influenza subtypes.

### Compliance Assessment

To assess compliance with isolation and quarantine recommendations, we interviewed all 66 households in which at least 1 person with pandemic influenza was isolated, 32 NSW households with at least 1 person with seasonal influenza, and 45 randomly selected quarantined NSW passengers. (NSW passengers were selected as a convenience sample.) Interviews were conducted by experienced public health interviewers who used a standardized questionnaire.

### Laboratory Investigation

NAT detection of pandemic influenza virus was performed by using real-time RT-PCR with primers targeting the hemagglutinin gene of the pandemic influenza virus provided by the Centers for Disease Control and Prevention and following recommended protocol or by using an in-house pandemic influenza virus–specific real-time RT-PCR. Seasonal influenza virus was identified by using a 2-target RT-PCR containing primers targeting pandemic and seasonal influenza virus strains (Unité de Génétique Moléculaire des Virus Respiratoires, Institut Pasteur, Paris, France) or a commercial influenza A subtyping assay (*Easy*-Plex Influenza profile 6; AusDiagnostics, Sydney, NSW, Australia).

### Statistical Analyses

We analyzed data by using Epi Info version 3.5.1 (www.cdc.gov/epiinfo). Relative risks were used to compare age (as a categorical variable split into 7 groups), sex, and place of residence. Fisher exact test results were used for cell sizes <5. A Mantel-Haenszel value of p<0.05 was considered significant. χ^2^ tests were used to compare proportions. To compare the rates of pandemic and seasonal influenza infection in childcare attendees, the number of sessions a child attended while susceptible were summed, and cases per child-sessions at risk and exact Poisson confidence intervals were calculated. Nonoverlapping confidence intervals were considered significantly different.

## Results

A total of 1,970 passengers and 734 crew members were on cruise A. Median age of passengers was 46 years (range 1–94 years), 57% were female, and most were from Australia (Table 1). Median age of crew members was 31 years (range 19–62 years), and most were born overseas (not in Australia). ILI developed in 13 (0.7%) passengers who sought medical attention during the cruise; and influenza A results from point-of-care testing were positive for 6. NAT of samples from persons who were sick during the cruise or during the 7 days after disembarkation showed positive pandemic influenza virus results for 76 (3.9%), positive seasonal influenza results for 98 (5.0%), and positive co-infection results for 2 (0.1%). ILI in the 7 days before disembarkation was reported by 15 (2.0%) crew members; NAT showed positive pandemic influenza results for 3 crew members and positive seasonal influenza results for none. These crew members were isolated on shore. The remaining 719 crew members were given oseltamivir prophylaxis and continued to work during cruise B; among these, 5 reported ILI (all within 24 hours of cruise B departing), and 3 had positive NAT results for pandemic influenza virus. Therefore, 20 (2.7%) crew members from cruise A reported ILI, and 6 (0.8%) of these had positive pandemic influenza test results; none had positive seasonal influenza test results. Given the relatively low attack rate for the crew, we focused further investigation on the passengers, among whom the attack rate for pandemic influenza was highest for children 3–6 years of age, followed by children 7–12 years of age. For seasonal influenza, the attack rate was similar among children in all age groups (Table 1).

**Table 1:** Demographics for passengers with influenza after 10-day cruise that departed Sydney, NSW, Australia, on May 16, 2009*

Demographic	No. (%) passengers, n = 1,970	Confirmed pandemic (H1N1) 2009, n = 78				Confirmed influenza A (H3N2), n = 100		
		No. (%)	RR (95% CI)	p value		No. (%)	RR (95% CI)	p value
Age group, y								
<3	13 (1)	0	0	1.00		1 (1)	1.52 (0.23–10.16)	0.67
3–6	48 (2)	20 (26)	17.43 (10.45–29.09)	<0.001		4 (4)	1.64 (0.62–4.36)	0.32
7–12	119 (6)	13 (17)	4.57 (2.40–8.69)	<0.001		6 (6)	1.00 (0.44–2.27)	0.99
13–18	114 (6)	2 (3)	0.73 (0.18–3.06)	1.00		5 (5)	0.87 (0.35–2.12)	0.75
19–35	369 (19)	18 (23)	2.04 (1.13–3.70)	0.020		19 (19)	1.02 (0.61–1.69)	0.95
36–65	1,046 (53)	25 (32)	Referent	–		53 (53)	Referent	–
>65	261 (13)	0	0.00 (undefined)	0.005		12 (12)	0.91 (0.50–1.70)	0.76
Sex								
M	842 (43)	35 (45)	Referent			47 (47)	Referent	
F	1,128 (57)	43 (55)	0.92 (0.59–1.42)	0.70		53 (53)	1.19 (0.60–1.2)	0.38
Residence								
NSW	1,135 (58)	47 (60)	Referent	–		62 (62)	Referent	–
Victoria	433 (22)	11 (14)	1.12† (0.72–1.74)	0.63		16 (16)	0.83† (0.56–1.24)	0.36
QLD	165 (8)	10 (13)				12 (12)		
SA	109 (6)	4 (5)				4 (4)		
WA	54 (3)	0				4 (4)		
ACT	39 (2)	3 (4)				1 (1)		
Tasmania	12 (1)	1 (1)				0		
NT	2 (0)	0				0		
Not Australia	7 (0)	2 (3)				1 (1)		
Unknown	14 (1)	0				0		


*Diagnosis received during 7-day period after the cruise. RR, relative risk; CI, confidence interval; –, not applicable; NSW, New South Wales; QLD, Queensland; SA, South Australia; WA, Western Australia; ACT, Australian Capital Territory; NT, Northern Territory. †Relative risks compared NSW residents with non–NSW residents. Two co-infected case-patients have been counted in both influenza categories. The 2 case-patients with positive results from point-of-care testing on board but no further subtyping results are excluded from this table.

### Symptoms and Severity of Illness

In total, 2 (3%) patients with pandemic influenza and 8 (8%) patients with seasonal influenza were hospitalized (p = 0.16); none died. Among the 50 passengers with the pandemic strain and 50 with the seasonal strain who were interviewed, symptoms were similar, although coryza was reported significantly more often by those with pandemic influenza (Table 2). Duration of illness was similar for passengers with either strain, but a higher proportion of seasonal influenza patients reported that illness was severe enough to limit their activities.

**Table 2:** Clinical data for 100 passengers with influenza after 10-day cruise that departed Sydney, New South Wales, Australia, on May 16, 2009

Clinical data	Pandemic (H1N1) 2009, no. (%), n = 50*	Influenza (H3N2), no. (%), n = 50†	p value
Cough	46 (92)	48 (96)	0.40
Fever (self-reported or measured)	39 (78)	34 (68)	0.26
Coryza	39 (78)	28 (56)	0.019
Fatigue	28 (56)	30 (60)	0.68
Sore throat	27 (54)	31 (62)	0.42
Headache	21 (42)	28 (56)	0.16
Myalgia	19 (38)	23 (46)	0.42
Dyspnea	12 (24)	10 (20)	0.63
Vomiting	5 (10)	4 (8)	0.73
Diarrhea	3 (6)	7 (14)	0.18
Severity of illness			
Limited activities	19 (38)	29‡ (58)	0.011
Antiinfluenza treatment	12 (24)	19§ (42)	0.06

*Patient median age 25.8 years (range 3–53 years); 20 male and 30 female; median duration of illness 5.0 days (range 0–17 days). †Patient median age 32.4 years (range 3–82 years); 25 male and 25 female; median duration of illness 7.0 days (range 1–35 days). Duration data from 45 reinterviewed case-patients. Excludes 5 case-patients for whom symptom data was collected at the time of testing but for whom laboratory results confirming influenza subtype H3N2 were not available at the time of reinterview. ‡Data from 45 reinterviewed case-patients. Excludes 5 case-patients for whom symptom data was collected at the time of testing but for whom laboratory results confirming influenza subtype H3N2 were not available at the time of reinterview. §Treatment given with 48 hours of symptom onset.

### Epidemiologic Investigation

According to date of symptom onset, the pandemic influenza outbreak began in the childcare center on May 18, which was 2 days after embarkation, and peaked on May 25, the final day of cruise A. The first reported seasonal influenza case was in an adult whose symptoms began on May 17; the second, seemingly unrelated, infection developed in a childcare attendee on May 21. The number of seasonal influenza cases also peaked on May 25 (Figure).

**Figure:**
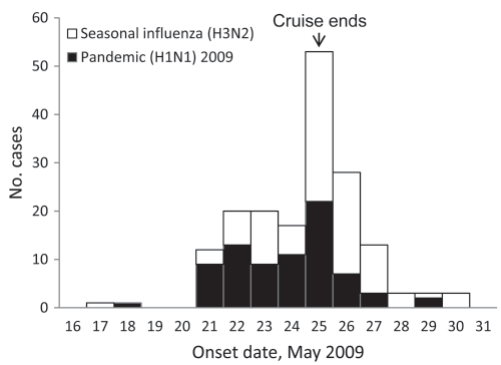
Date of onset of first symptoms for cruise ship passengers, by influenza subtype. Excludes 1 influenza A (H3N2) case-patient for whom onset date was unavailable and 1 pandemic (H1N1) 2009 case-patient and 2 influenza A (H3N2) case-patients who were asymptomatic but whose laboratory test results were positive.

### Childcare Center Investigation

Of the 48 passengers 3–6 years of age, 45 (94%) attended childcare. Among these 45 were 8 pairs of siblings. One child received seasonal influenza vaccine in 2008 and no child received the vaccine in 2009. The first case of pandemic influenza was in a child from Victoria, Australia, in whom symptoms developed on the third day of cruise A. The child attended childcare for 4 sessions while infectious. After the index case was identified, 19 additional cases of pandemic influenza were identified (including in 2 sets of siblings: 2 related children in whom symptoms developed at the same time and in 1 child in whom ILI developed 2 days after symptom onset in her sibling); all but 1 had attended the childcare while a known infectious case-patient was present. The first child for whom seasonal influenza was diagnosed had attended childcare the afternoon and evening before symptom onset on May 21 and for 6 sessions while symptomatic. Subsequently, an additional 3 unrelated cases of seasonal influenza were identified among childcare attendees. The 3 children who did not attend childcare remained healthy.

Among the 45 childcare attendees, NAT results for pandemic influenza were positive for 18, for seasonal influenza were positive for 2, and for both were positive for 2. ILI developed in an additional 10 children, but these children had negative influenza results by NAT; ILI developed in another 6 children who were not tested. Of these 16 children, 8 had traveling companions with positive pandemic influenza virus results and 2 had travelling companions with positive seasonal influenza virus results. The remaining 7 children remained asymptomatic. Of the 45 children who attended childcare, 44 attended concurrently with an infectious pandemic influenza case-patient and 43 attended concurrently with an infectious seasonal influenza case-patient. Considering the number of sessions attended by susceptible children, we determined that the risk for pandemic influenza infection was significantly higher (19 cases from 344 sessions = 0.055 child-sessions at risk, 95% confidence interval [CI] 0.033–0.086) than was the risk for seasonal influenza (3 cases from 279 sessions = 0.011 child-sessions at risk, 95% CI 0.002–0.031).

### Secondary Attack Rates for Pandemic Influenza

A total of 66 pandemic influenza case-patients in 53 cabins were infectious while on cruise A. Excluding the co-primary case-patients, 91 passengers shared a cabin with an infectious primary case-patient. Of these 91 passengers, symptoms developed in 50 (55%). Of these 50 case-patients, 34 were tested and 12 (35%) had positive pandemic influenza results. The secondary attack rate for those <12 years of age (16/21) was significantly higher than for those >12 years of age (34/70) (76% vs. 49%; p = 0.03). Of the 66 case-patients, 1 received oseltamivir treatment within 48 hours of symptom onset. Information about provision of oseltamivir prophylaxis was available for 34 (83%) of 41 asymptomatic contacts. Of these, 3 (75%) of 4 children <12 years of age and 17 (57%) of 30 children >12 years of age began receiving antiviral drug prophylaxis within 7 days of their first exposure to pandemic influenza virus. Of these 20, only 1 received prophylactic drug within 3 days of first exposure to the pandemic strain. Despite being asymptomatic, 11 (27%) of 41 passengers underwent laboratory testing and were negative for pandemic influenza virus by NAT.

### Isolation and Quarantine

After disembarking, patients with pandemic and seasonal influenza were isolated in 149 discrete (family or household-like) groups. Of the 98 (66%) interviewed, 37% reported that they were first made aware of the need for isolation through media reports, 27% by their treating doctor, 26% by public health staff, 6% by the ship’s staff, and 5% by fellow passengers. Of the 45 quarantined passengers interviewed, 52% were initially informed of the need for quarantine through media reports, 25% by work or school colleagues, 11% from the ship’s staff, 7% from a friend or relative, and 5% from public health staff. All influenza case-patients reported that they had obeyed isolation requirements, and 43 of 45 quarantined passengers reported that they had remained in quarantine for 7 days after disembarkation. Of the 2 passengers who did not follow quarantine requirements, 1 reportedly attended work by private vehicle and cancelled all other outings; the other denied knowledge of the requirements.

### Further Virus Transmission

Three secondary pandemic influenza infections among family contacts of case-patients from cruise A were identified; a subsequent case-patient was identified as a contact of 1 person who had secondary infection. Other than these cases, no evidence of transmission to the community or to passengers of cruise B was found.

## Discussion

We identified dual outbreaks of pandemic and seasonal influenza among passengers on a cruise ship. Cruise ships provide ideal conditions for rapid spread of respiratory viral illnesses (e.g., many persons living closely together, frequently interacting in enclosed and partially enclosed environments, and often originating from both hemispheres). Although infections spread rapidly among passengers and to some crew members during the cruise, further spread to the community and the next cruise was avoided through intensive disease control measures.

After identification of the outbreak, it became apparent that undetected local transmission of pandemic influenza virus was occurring in Victoria before cruise A (*8*) and that the virus was probably introduced to the ship by the index case-patient from Victoria. The pandemic virus spread rapidly among other childcare attendees and their close contacts and to other passengers and crew. Seasonal influenza virus was the predominant influenza virus circulating in NSW before the appearance of pandemic influenza virus (NSW Health, unpub. data).

The cocirculation of both strains in the childcare center provided a unique opportunity to compare attack rates. The pandemic strain seems to have spread among children more readily than the seasonal strain. This difference in transmissibility could have resulted from innate differences in the viruses themselves or from a level of immunity from past infection with the seasonal strain. Consistent with findings in other studies, the symptoms of pandemic and seasonal influenza were similar (*9**–**12*). After adjusting for underlying medical conditions, we found that hospitalization rates and activity-limiting effects were higher for case-patients with seasonal than with pandemic influenza; however, this finding may be explained in part by differences in the age-specific attack rates. The secondary attack rate for pandemic influenza among cabin contacts of 55% was higher than that reported for household contacts (*13**,**14*), despite a small proportion of these persons having received antiviral drug prophylaxis, and may reflect the close living arrangements in a ship’s cabin.

The intense passenger follow-up enabled us to assess the sensitivity of the ship’s medical clinic for identifying influenza cases. Before this outbreak, ships had active containment measures in place to minimize the spread of seasonal influenza, including use of point-of-care influenza testing for patients seeking treatment for ILI and oseltamivir treatment and isolation to reduce further spread. Our active case-finding efforts identified 79 influenza cases on cruise A, yet the ship’s clinic identified only 6 (8%) of these. Despite enhanced community awareness of the emerging pandemic, the ship’s medical clinic staff underestimated the case count by 13-fold. The number of passengers who sought treatment at the ship’s medical clinic does not accurately reflect the extent of the influenza outbreak on board, possibly because the decision to seek treatment may have been influenced by a number of factors including cost, severity of symptoms, and unwillingness to be isolated while on holiday.

Our investigation had several limitations. First, the case definition depended on NAT detection of virus in clinical samples, which may have resulted in misclassification of cases. Second, although the epidemiology is consistent with the first cases of pandemic influenza appearing in the childcare center, undetected or asymptomatic infected passengers or crew could have carried the viruses onto the ship. However, this scenario is unlikely because the symptoms developed in the index case-patient 2 days after embarkation. Third, although most ill passengers were interviewed within 2 days after onset of illness, interviews about severity, length of illness, and the experience in isolation and quarantine were conducted some weeks later, introducing possible recall bias. Fourth, although 2 cases of co-infection were detected, only the first 2 pandemic influenza–positive specimens from childcare attendees were subtyped for other influenza A subtypes; it is possible that some of the remaining pandemic influenza case-patients were also infected with seasonal influenza. Fifth, some of the remaining pandemic influenza case-patients may have been co-infected. Sixth, the secondary attack rate for cabin contacts may be an overestimate because passengers with negative NAT results were not tested for other respiratory infections, and passengers with onset of symptoms >24 hours after symptoms developed in a cabinmate were assumed to be secondary, rather than co-primary cases.

Mathematical modeling suggests that containment of influenza is possible if appropriate resources are devoted. In some countries, isolation and quarantine measures have been used in response to severe acute respiratory syndrome (*15**–**18*), but these measures have rarely been used for influenza control. In the influenza outbreaks reported here, direct follow-up of passengers in isolation and quarantine, supported by intense media coverage, resulted in a high degree of compliance and successful outbreak containment. Additionally, providing oseltamivir prophylaxis for crew members may have contributed to the successful containment of the infection during cruise B. Although the robust application of containment measures can stop the spread of novel influenza viruses, public health resource requirements are labor-intensive and expensive and may not be sustainable except for the most virulent of pandemic viruses.
